# Quantitative Trait Loci Mapping for Lameness Associated Phenotypes in Holstein–Friesian Dairy Cattle

**DOI:** 10.3389/fgene.2019.00926

**Published:** 2019-10-04

**Authors:** Enrique Sánchez-Molano, Veysel Bay, Robert F. Smith, Georgios Oikonomou, Georgios Banos

**Affiliations:** ^1^The Roslin Institute and R(D)SVS, University of Edinburgh, Easter Bush, Edinburgh, United Kingdom; ^2^Institute of Infection and Global Health, University of Liverpool, Liverpool, United Kingdom; ^3^Bandirma Sheep Research Institute, The Ministry of Agriculture and Forestry, Balikesir, Turkey; ^4^Institute of Veterinary Science, University of Liverpool, Leahurst Campus, Liverpool, United Kingdom; ^5^The Roslin Institute Building, Scotland’s Rural College, Easter Bush, Edinburgh, United Kingdom

**Keywords:** Lameness, GWAS, welfare, regional heritability mapping, QTL

## Abstract

Lameness represents a significant challenge for the dairy cattle industry, resulting in economic losses and reduced animal health and welfare. The existence of underlying genomic variation for lameness associated traits has the potential to improve selection strategies by using genomic markers. Therefore, the aim of this study was to identify genomic regions and potential candidate genes associated with lameness traits. Lameness related lesions and digital cushion thickness were studied using records collected by our research team, farm records, and a combination of both. Genome-wide analyses were performed to identify significant genomic effects, and a combination of single SNP association analysis and regional heritability mapping was used to identify associated genomic regions. Significant genomic effects were identified for several lameness related traits: Two genomic regions were identified on chromosome 3 associated with digital dermatitis and interdigital hyperplasia, one genomic region on chromosome 23 associated with interdigital hyperplasia, and one genomic region on chromosome 2 associated with sole haemorrhage. Candidate genes in those regions are mainly related to immune response and fibroblast proliferation. Quantitative trait loci (QTL) identified in this study could enlighten the understanding of lameness pathogenesis, providing an opportunity to improve health and welfare in dairy cattle with the addition of these regions into selection programs.

## Introduction

Lameness is a complex trait defined as an abnormal stance or gait of the animal that results from disorders of the locomotor system. In dairy cattle, lameness is one of the most important health conditions together with impaired fertility and mastitis (Green et al., 2002; Buitenhuis et al., 2007; Cha et al., 2010), and causes important economic losses and reduced animal health and welfare (Huxley, 2013).

Many lameness cases are associated to various infectious and non-infectious diseases (Green et al., 2002; Van Der Waaij et al., 2005; Bicalho and Oikonomou, 2013), resulting in painful foot lesions such as sole ulcers, white line lesions, sole haemorrhages, interdigital hyperplasia, and others. Previous studies have shown that both animal genetic and management factors contribute to the development of these diseases (Olmos et al., 2009; Van Der Linde et al., 2010; Swalve et al., 2014). The existence of genetic variation underlying lameness-associated traits has been previously demonstrated using pedigree data analyses, with heritability estimates ranging from 0.06 to 0.52 (Boettcher et al., 1998; Zwald et al., 2004; Koenig et al., 2005; Laursen et al., 2009; Schopke et al., 2015). Furthermore, susceptibility to certain non-infectious foot lesions is also associated with morphological hoof traits such as the thickness of the digital cushion, a complex, force dissipating, subcutaneous tissue located under the distal phalanx (Bicalho et al., 2009).

While reducing the incidence of lameness is one of the main objectives for the dairy cattle industry, current evaluations are based on observational scores such as claw health status data, lameness and mobility scores, conformational traits, and data collected by automated sensors (Heringstad et al., 2018). All this information is obtained once the animal has started to show symptoms of lameness, thus not being available early in life and also showing relatively low heritabilities (Buitenhuis et al., 2007). Therefore, the identification of genomic regions and genes associated to lameness and lameness associated traits could strongly improve selection strategies by providing genomic information to make early breeding decisions and potentially informing more accurate genomic-based selection programmes.

Few studies have addressed traits associated with lameness using a genomic approach. The largest study (Malchiodi et al., 2018) grouped lameness-associated lesions into two categories, infectious and non-infectious, and used single nucleotide polymorphism (SNP) data to identify several genomic regions with candidate genes linked to immune system, morphogenesis, and cell proliferation. Other studies used microsatellite data (Buitenhuis et al., 2007) to identify lameness-associated regions and SNP data (Scholey et al., 2012; Swalve et al., 2014) to identify regions associated to digital dermatitis and sole hemorrhage. The relatively high number of identified regions together with the complex aetiology of lameness seems to support a potential polygenic architecture with many genes influencing the different biological factors involved. Therefore, it is necessary to study the different types of lesions separately in order to identify particular and common genomic regions that contribute to the main condition phenotype.

The objective of the present study is to perform genome-wide analyses to identify regions and candidate genes, and understand the genetic basis of a wide range of lameness-related traits. This knowledge may inform genetic improvement schemes aiming to reduce prevalence of dairy cattle lameness. Digital cushion thickness (DCT) measurements are studied here for the first time from a genomic perspective.

## Materials and Methods

### Animals and Phenotypes

Ethical approval for the study was granted by the University of Liverpool Research Ethics Committee. ASPA regulated procedures were conducted under a Home Office License (Reference Number: PPL 70/8330).

The study included a total of 554 Holstein–Friesian cows in lactation 0–8 from three different farms. Data sources were from a combination of previous research projects and routinely kept farm records. The recorded lameness-causing foot lesions were digital dermatitis, sole ulcer, white line disease, sole haemorrhage, and interdigital hyperplasia. Cases were defined following the ICAR Claw Health Atlas definitions (Egger-Danner et al., 2015).

Farm phenotypic records for presence (1) or absence (0) of these lesions were extracted from the farm database for all these animals (single record per animal) from May 2006 to October 2017 using TotalVet software (Sum-IT). In addition, 475 cows were individually monitored for the same lesions by a research team led by an experienced veterinarian during three separate time intervals between December 2014 and October 2017. DCT measurements were taken using ultrasonography between December 2014 and January 2016 (1^st^ research interval), and between October 2016 and August 2017 (2^nd^ research interval). A number of cows were also followed during the period between August 2017 and October 2017, but no DCT measurements were obtained (3^rd^ research interval). Lameness lesions were recorded by the veterinarian for 88 animals between December 2014 and January 2016, and for 337 animals between October 2016 and October 2017; 50 cows had records from both these two different time intervals. DCT measurements and recording of lameness-causing lesions were performed at three time points around animals’ calving: 3–4 weeks before the expected calving, the first week after calving, and approximately 8 weeks after calving. Seventy-nine animals only had lameness lesion records obtained from the farms’ records. Research and farm records were analysed both separately and combined. For the latter, animals were considered as affected when at least one of the available records (research or farm) indicated presence of the lesion. All these data are summarised in **Table 1**.

**Table 1:** Number of cows enrolled *per* farm and source of data collection.

Farm	n	Source of data collection	n
**1**	307	0	79
		1	88
		2	81
		3	9
		4	45
		5	5
**2**	135	2	74
		3	61
**3**	112	2	112

Source of data collection: 0 = farm records only, 1 = research data collected during 1st research interval (December 2014 and January 2016), 2 = research data collected during 2nd research interval (October 2016 and August 2017), 3 = research data collected during 3rd research interval (August 2017 and October 2017), 4 = research data collected during 1st and 2nd research intervals, 5 = research data collected during 1st and 3rd research intervals.

Cows were restrained in a foot trimming crush for the measurement of DCT and the recording of lameness-causing foot lesions. Measurement of DCT was performed using an Easi-Scan ultrasound machine (sonographic B-mode, BCFTM Technology, UK) equipped with a linear probe 5–8 MHz. All measurements of DCT were undertaken at the midline, on the lateral claw of the hind left foot. To measure the DCT, the foot was cleaned and loose horn was removed with a hoof knife. Sole contact with the transducer was made using ultrasound gel (Ultrasound Gel, Henry Schein) and a gel standoff (Flexi gel standoff, BCFTM Technology, UK). After freezing the image on the ultrasound monitor (Easi-Scan Ultrasound Remote Display, BCFTM Technology, UK), measurements were taken to the nearest millimetre. The DCT was measured just cranial to the tuberculum flexorum of the pedal bone at the typical sole ulcer site. The distance from the inner margin of the sole (identified as a thin echogenic line) to the distal edge of the pedal bone (identified as a thick echogenic line) was assessed.

### DNA Sampling, Extraction, and Genotyping

Blood samples were collected from the tail vein of each cow in vacutainer tubes containing EDTA. Genomic DNA was extracted from buffy coat samples using the QIAamp DNA Blood MiniKit from Qiagen. Extracted DNA samples were quantified using a NanoDrop and stored at -20°C. Initially, 266 cows were genotyped using the Affymetrix Axiome bovine 54K SNP array. The Illumina BovineSNP50 bead chip containing 53,714 SNP was used to genotype the rest of the animals. Genotype data obtained from the Affymetrix array were converted to the Illumina chip format by selecting common SNPs using concordant strand assignment and identified allelic calls before further analyses were conducted.

### Sample and Genotype Quality Control

Quality control was performed using PLINK (Purcell et al., 2007) in order to assess both sample and marker quality. A minimum genotype call rate of 95% was applied, removing SNPs with low genotyping quality. Further quality control on the markers removed those with low minor allele frequency (MAF < 0.01) and showing strong deviations from Hardy-Weinberg equilibrium (threshold of 1.45E-6 calculated genome-wide by applying a Bonferroni correction to obtain a nominal *P*-value of 0.05). The final genotype call rate after genotype quality control was 98.7%. Additional quality control of samples was performed by removing individuals with poor genotype quality (sample call rate lower than 95%). All these quality control procedures resulted in a final dataset of 549 animals genotyped for 34,658 SNPs with positions assigned according to the UMD 3.1 assembly.

### Population Structure

Principal component analyses of the genotyped animals were performed using GEMMA (Zhou and Stephens, 2012), replacing missing genotypes with the average genotype. Visual exploration showed a relatively light population structure not explained by any of the descriptive factors (e.g., farm, parity number, lactation, etc.). Therefore, the genomic relationship matrix (GRM) among animals was fitted in all ensuing statistical models of analysis as a random polygenic effect to account for any potential inflation effects caused by population structure.

### Estimation of Variance Components

Estimates of the variance components for each individual trait were obtained by fitting the following model using REACTA (Cebamanos et al., 2014):

(1) y=Wα+Ζu+ε

where **y** represents the vector of phenotypes, **W** is an incidence matrix, **α** is the vector of associated fixed effects, **Z** is the design matrix for the vector **u** of random polygenic effects [distributed as a multivariate normal distribution MVN(0,V_g_**G**) with **G** being the GRM and V_g_ the genetic variance of the trait], and **ε** represents the vector of residual errors [distributed as MVN(0,V_e_**I**) with **I** being the identity matrix and V_e_ the residual variance]. The significance of the genomic (polygenic) effect (*P* = 0.05) was assessed using the likelihood ratio test statistic to compare a model that fits the effect against the base model that excludes it.

Fixed effects used in model (1) were tested previously using Wald tests in ASReml 4 (Gilmour et al., 2009), fitting a logit model for disease traits and a linear model for DCT records, and following a backward elimination approach. After performing analyses for all traits, concordant models were chosen incorporating as fixed effects: i) for the disease research records: farm (3 levels), parity number at recording (three levels, 1, 2, and ≥3), and research interval (five levels, grouped as 1 = interval 1, 2 = interval 2, 3 = interval 3, 4 = intervals 1 and 2, 5 = intervals 1 and 3); ii) for the farm and combined disease records: farm (as before), lactation number at the end of study (four levels 0, 1, 2, and >3), and interval (as before); and iii) for the DCT records: farm (as before), parity number (as before), and assessor (six levels).

### Genome-Wide Association Analysis

Individual SNP association analyses were performed in those traits with a significant genomic effect from model (1) using GEMMA (Zhou and Stephens, 2012). The linear mixed model was:

(2) y=Wα+xβ+Zu+ε

where ***x*** represents the vector of genotypes (coded as 0/1/2) and β is the regression coefficient of the phenotype on the genotypes; all other effects are as described in model (1). The statistical significance of the regression coefficient was assessed using a Wald test. When determining the significant thresholds, a Bonferroni correction was performed for multiple testing due to the number of markers, but not for multiple traits. This resulted in a genome-wide significant threshold (*P*  =  0.05) defined at *P*  =  1.44E-6 [-log10(*P*)  =  5.84] and a suggestive threshold (one false positive *per* genome scan, *P*  =  1) defined at *P*  =  2.89E-5 [-log10(*P*)  =  4.54].

Despite including the polygenic effect in the model, genotyping errors or other artefacts such as cryptic population structure may inflate test statistics. Therefore, to account for any potential remaining inflation, the ratio of the median of the empirically observed distribution of the test statistic to the expected median (inflation factor λ) was used for correction, following the method described by Amin et al. (2007), which assumes that the inflation is constant across the genome.

### Regional Heritability Mapping

Under the regional heritability mapping approach, the genome was divided into non-overlapping windows of 20 consecutive SNPs. The following model was used in REACTA (Cebamanos et al., 2014):

(3) $$y=W\alpha +Xu_{\left(i\right)}+Zu_{\left(-i\right)}+\varepsilon$$

where **X** and **Z** are the corresponding design matrices for the effects **u_(i)_** of the corresponding region *i* {distributed as MVN[0,V_g(i)_**G_(i)_**], with V_g(i)_ and **G_(i)_** being the genomic variance and the GRM corresponding to the SNPs in the *i^th^* region, respectively} and **u**_(-i)_ of the genome (polygenic effect) excluding the region *i* {distributed as MVN[0,V_g(-i)_**G_(-i)_**] with V_g(-i)_ and **G_(-i)_** being the genetic variance and the GRM corresponding to all SNPs other than those on the region *i*, respectively}.

The significance of the region effect was assessed using the likelihood ratio test statistic. A total of 1,733 regions were analysed, leading to a genome-wide significant threshold (*P* = 0.05) defined at *P*  =  2.89E-5 with Bonferroni correction for multiple regions [-log10(*P*)  =  4.54] and a suggestive threshold (one false positive *per* genome scan) defined at *P*  =  5.77E-4 [-log10(*P*)  =  3.24]. As with the genome-wide association analyses, a correction by the inflation factor λ was applied to account for any remaining inflation after fitting the polygenic effect in the model.

## Results

### Descriptive Statistics

The number of cows enrolled *per* farm and *per* period of data collection is presented in **Table 1**. Incidences of each lameness-inducing foot lesion *per* farm are summarised in **Table 2**. Number of cows *per* farm with no, one, two, or three or more of the recorded lesions are presented in **Table 3**.

**Table 2:** Incidence of digital dermatitis (DD), interdigital hyperplasia (IH), sole haemorrhage (SH), sole ulcer (SU), and white line disease (WLD) *per* farm.

Farm	SU	SH	WLD	DD	IH
**1**	0.26	0.28	0.32	0.40	0.23
**2**	0.27	0.42	0.24	0.57	0.07
**3**	0.11	0.09	0.07	0.46	0.12

**Table 3:** Number of cows *per* farm with zero, one, two, and three or more recorded lesions (digital dermatitis, interdigital hyperplasia, sole haemorrhage, sole ulcer, and white line disease)

Farm	Number of lesions *per* cow	n
**1**	0	86
	1	82
	2	69
	3+	70
**2**	0	21
	1	55
	2	31
	3+	28
**3**	0	44
	1	48
	2	14
	3+	6

### Population Structure

**Figure 1:**
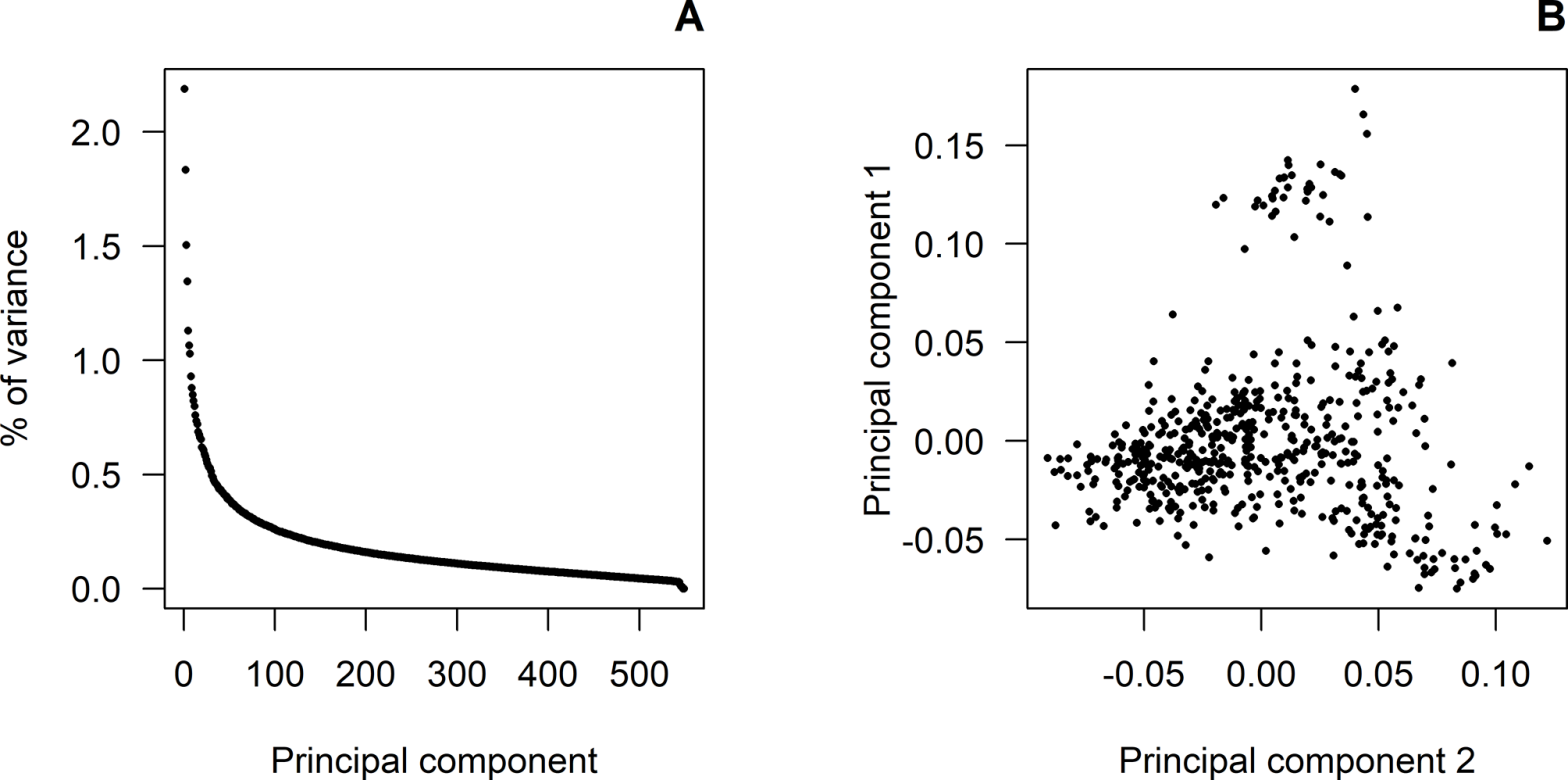
Principal component analyses: **(A)** shows the proportion of variance (%) corresponding to the decomposition of the genomic relationship matrix for each of the principal components. **(B)** shows the population structure according to the first and second principal components.

Figure 1A shows the eigenvalues corresponding to the principal component analysis performed on the GRM of the genotyped animals. The first seven principal components accounted for about 10% of the total variance, with the first three components explaining 2.19%, 1.83%, and 1.50%, respectively. Figure 1B shows a light population structure mainly due to the first principal component. No population attributes were available that could explain this. Therefore, a polygenic effect based on GRM was fitted to account for this light population structure.

### Full Genomic Variance Analysis

**Table 4** shows the variance component estimates in the observed scale for those traits with a significant genomic effect (*P* < 0.05) based on the likelihood ratio test. Heritability estimates for the disease traits range from 0.129 (white line disease in combined records) to 0.516 (interdigital hyperplasia in research records), corresponding to genomic variances in the range of 0.008–0.067. Heritability for DCT at calving was moderate (0.228), corresponding to a genetic variance of 0.549.

**Table 4:** Estimates of heritability and variance components for traits with a significant (*P* < 0.05) genomic effect.

	Trait	h^2^	Vg	Ve	*P*	N
**DCT records**	**DCT_fresh**	0.228 ± 0.119	0.549 ± 0.298	1.854 ± 0.290	6.69E-3	360
	**DD**	0.185 ± 0.093	0.043 ± 0.022	0.189 ± 0.023	5.55E-3	469
**Research records**	**IH**	0.516 ± 0.105	0.067 ± 0.016	0.063 ± 0.012	1.05E-8	469
	**SH**	0.204 ± 0.099	0.035 ± 0.017	0.135 ± 0.017	1.25E-2	469
	**DD**	0.196 ± 0.088	0.023 ± 0.011	0.094 ± 0.011	5.89E-3	549
**Farm records**	**SU**	0.290 ± 0.091	0.037 ± 0.013	0.092 ± 0.011	6.29E-5	549
	**IH**	0.131 ± 0.076	0.008 ± 0.005	0.054 ± 0.005	1.85E-2	549
	**DD**	0.201 ± 0.080	0.046 ± 0.019	0.183 ± 0.019	2.56E-4	549
	**SU**	0.350 ± 0.096	0.057 ± 0.017	0.106 ± 0.015	8.24E-6	549
**Combined records**	**WLD**	0.129 ± 0.081	0.020 ± 0.013	0.135 ± 0.014	2.83E-2	549
	**IH**	0.368 ± 0.088	0.047 ± 0.013	0.081 ± 0.011	4.42E-8	549
	**SH**	0.143 ± 0.083	0.024 ± 0.014	0.142 ± 0.015	3.19E-2	549

Genomic heritabilities (h^2^) and genomic (Vg) and residual variances (Ve) estimated together with their standard errors. P-values (P) for the significance of the genomic effect and the number of total records (N). Digital cushion thickness at calving (DCT_fresh), digital dermatitis (DD), interdigital hyperplasia (IH), sole haemorrhage (SH), sole ulcer (SU), and white line disease (WLD).

### Genome-Wide Association Analysis

Two genome-wide significant SNPs (for interdigital hyperplasia and digital dermatitis in farm records) and 19 genome-wide suggestive SNPs were detected in the genome-wide association analyses (**Table 5**). After performing the correction by the inflation factor, all λ estimates ranged from 1.002 to 1.033, thus implying the absence of any significant inflation in the test estimates.

**Table 5:** Significant SNP from the genome-wide association analyses.

	Trait	BTA	Position (BP)	Beta coef.	MAF	*P*
**Research records**	**DD**	3	70931186	0.380 ± 0.086	0.038	1.23E-5
		20	30216498	0.166 ± 0.037	0.276	7.74E-6
	**IH**	11	99952182	0.180 ± 0.042	0.098	2.54E-5
	**SH**	2	4958110	0.170 ± 0.038	0.171	1.00E-5
**Farm records**	**DD**	3	70931186	0.275 ± 0.056	0.038	1.32E-6
		7	28258117	0.322 ± 0.074	0.020	1.97E-5
		19	10140328	0.264 ± 0.062	0.027	2.91E-5
		24	37354445	0.137 ± 0.031	0.147	1.32E-5
	**SU**	12	12612422	0.185 ± 0.043	0.084	2.82E-5
	**IH**	23	44153826	0.266 ± 0.048	0.027	4.83E-8
**Combined records**	**DD**	3	70931186	0.383 ± 0.080	0.038	2.17E-6
		3	90367814	0.224 ± 0.047	0.123	2.22E-6
		3	90523019	0.239 ± 0.053	0.096	7.42E-6
		20	30216498	0.165 ± 0.035	0.270	2.30E-6
	**WLD**	5	94496854	−0.106 ± 0.025	0.479	2.31E-5
		7	75190535	−0.122 ± 0.027	0.339	4.99E-6
		14	5883219	−0.148 ± 0.034	0.152	1.60E-5
	**IH**	2	23628756	0.157 ± 0.037	0.106	2.07E-5
		3	23764339	0.160 ± 0.037	0.119	1.61E-5
	**SH**	2	4958110	0.152 ± 0.035	0.165	1.93E-5
		21	46018333	0.262 ± 0.057	0.052	4.94E-6

Chromosome (BTA) and base pair position follow the UMD 3.1 assembly. Beta coefficient (minor allele substitution effect) and standard error, minor allele frequency (MAF), and P-value (P) for the beta coefficient. Digital dermatitis (DD), interdigital hyperplasia (IH), sole haemorrhage (SH), sole ulcer (SU), and white line disease (WLD).

MAFs ranged from 0.020 to 0.479, and most substitution effects were positive (with the exception of white line disease in the combined records), thus implying a positive effect of the minor allele against the disease. However, due to a possible Beavis effect (Xu, 2003), the provided effect sizes may be slightly overestimated.

DCT at calving and sole ulcer in the combined records did not provide any significant or suggestive SNP despite showing a significant genomic effect in the previous analyses.

### Regional Heritability Mapping and Concordant Regions

A significant region for interdigital hyperplasia was detected based on farm records, and 10 suggestive regions were detected for other traits using the regional heritability mapping approach (**Table 6**). The significant region detected on chromosome 3 for digital dermatitis using research records was also detected as suggestive for interdigital hyperplasia using the combined records.

**Table 6:** Significant genomic regions from the regional heritability mapping analyses.

	Trait	BTA	Position (BP)	*P*
**Research records**	**DD**	22	34216267–36047120	4.14E-4
		22	31087678–32538832	4.36E-4
**Farm records**	**DD**	3	70077512–71882823	3.40E-4
		25	34887253–35853810	2.61E-4
	**SU**	25	3126438–4354023	8.03E-5
	**IH**	23	43151282–44458259	2.28E-6
**Combined records**	**SU**	25	3126438–4354023	1.86E-4
	**WLD**	14	6850767–7718808	5.43E-4
	**IH**	3	22069239–23764339	1.35E-4
		3	70077512–71882823	5.94E-5
	**SH**	2	4587203–5640288	3.35E-4

Chromosome (BTA) and base pair position follow the UMD 3.1 assembly. P-value (P) for the region effect. Digital dermatitis (DD), interdigital hyperplasia (IH), sole haemorrhage (SH), sole ulcer (SU), and white line disease (WLD).

Four of the detected regions were concordant with suggestive/significant SNPs detected in the genome-wide association analyses (**Table 5** and **Supplementary Figure 1**), two of them detected from the farm records and another two from the combined farm and research records. In the farm records, the concordant regions detected both by genomic analyses explained 32.90% and 43.90% of the total genomic variance of digital dermatitis and interdigital hyperplasia, respectively. In the combined records, the concordant regions explained 10.53% and 11.29% of the total genomic variance of interdigital hyperplasia and sole haemorrhage, respectively. Again, caution must be exercised while assessing these findings because of possible overestimation due to a potential Beavis effect (Xu, 2003).

## Discussion

In the present study, two genome-wide association approaches were used to identify quantitative trait loci (QTL) affecting lameness related traits. Comparison of the results provided by individual SNP genome-wide association analyses and regional heritability mapping was performed to strengthen the evidence of the identified regions. QTLs were detected for digital dermatitis, interdigital hyperplasia, and sole haemorrhage.

Three sources of data were used in this study in order to identify genomic regions for lameness related diseases. Although using only research-confirmed records is expected to provide more accurate phenotypes than using farm records, the number of observations available was smaller, thus reducing the power to detect significant genomic effects. Similarly, using farm records provided a larger number of records spanning animals’ whole lifetime; these records however are potentially less accurate, thus leading to a low detection power and increase the chances of introducing misclassification bias. Impact of phenotypic errors in genomic analyses has been discussed before both in the context of human (Buyske et al., 2009) and animal data (Biffani et al., 2017). Furthermore, it has been shown previously that farm records could seriously under-record certain lesions (Heringstad et al., 2018) and this has also been the case with our dataset. The most powerful dataset available in the present study was the combination of farm and research records, which provided a larger number of records than research alone but more accurate compared to farm, thus leading to significant estimates of genomic effects for more traits.

Heritability estimates for some of these traits have been previously estimated using pedigree data (Koenig et al., 2005; Van Der Waaij et al., 2005; Gernand et al., 2012; Oberbauer et al., 2013; Van Der Spek et al., 2013; Malchiodi et al., 2017). Such estimates range from 0.07 to 0.4 for digital dermatitis, 0.10 to 0.39 for interdigital hyperplasia, and 0.04 to 0.17 for sole haemorrhage. Our heritability estimates were generally in concordance within these ranges, particularly considering the estimates obtained using the combined records. It has to be recognised that heritability estimates are presented in the observed scale (0–1) and, therefore, population parameters are dependent on the disease prevalence (Lee et al., 2011). However, it is expected that estimates will not vary widely when transformed to the liability scale (normal distribution), where the mean and variance are independent from the prevalence of the disease. In the case of digital dermatitis, the heritability observed for the combined records was 0.20, resulting in estimates between 0.21 and 0.30 on the liability scale when assuming a disease prevalence from 10% to 30% (Holzhauer et al., 2006). Similarly, the heritability observed for interdigital hyperplasia was 0.37, resulting in a heritability of 0.39 on the liability scale when assuming a prevalence of 1.3% (Solano et al., 2016).

Although genome-wide association and regional heritability mapping analyses revealed several QTLs independently, four QTLs were commonly reported by both approaches (**Tables 2** and **3** and **Supplementary Figure 1**). On chromosome 3, a suggestive region was associated with digital dermatitis in farm records, explaining 32.90% of the total genomic variance and being also suggestive of interdigital hyperplasia in the combined records. Two potential gene candidates are contained within this region: i) *FPGT* (fucose-1-phosphate guanylyl transferase) part of the L-fucose pathway, a key sugar in complex carbohydrates involved in cell-to-cell recognition, inflammation, and immune processes (Becker and Lowe, 2003); and ii) *TNNI3K* (serine/threonine-protein kinase TNNI3K), also associated with inflammation mechanisms (Wiltshire et al., 2011). Based on this function, we surmise that a candidate gene for lameness resistance may be found within this QTL.

On chromosome 23, a significant region was associated with interdigital hyperplasia in farm records, explaining 43.90% of the total genomic variance. Interdigital hyperplasia, also known as interdigital fibroma (Atkinson, 2013), results in a thickening of interdigital connective tissue causing fibroid tumours. Thus, a potential candidate gene found within this region is *EDN1* (endothelin-1), a vasoconstrictor associated with several cardiovascular diseases and inflammatory and fibrotic processes (Matsushima et al., 2004), acting as fibroblast mitogen in systemic sclerosis (Vancheeswaran et al., 1994), pulmonary fibrosis (Hocher et al., 2000), and hepatic fibrosis (Rockey and Chung, 1996).

On chromosome 3, another suggestive region was associated with interdigital hyperplasia using combined records, explaining 10.53% of the total genomic variance. This region includes several potential candidate genes, particularly *PHGDH* (D-3-phosphoglycerate dehydrogenase), an oxidoreductase that has been associated previously with pulmonary fibrosis (Hamanaka et al., 2017).

On chromosome 2, a suggestive region was associated with sole haemorrhage from the combined records, explaining 11.29% of the total genomic variance. With sole haemorrhage being related to impaired vascular system and cellular inflammatory reactions (Ossent and Lischer, 1998), a potential candidate gene within this region is *GPR17* (uracil nucleotide/cysteinyl leukotriene receptor). This gene is as a sensor molecule involved in traumatic, vascular, and inflammatory pathologies in the central nervous system (Boda et al., 2011), and is also related to vascular permeability and inflammatory processes as a regulator of the cysteinyl leukotriene 1 receptor response (Maekawa et al., 2009).

Most candidate genes within the detected genomic regions are related either to inflammatory processes or fibroblast proliferation, as expected due to the nature of the analysed traits. However, these are not independent processes, but linked networks where fibroblasts present complex biosynthetic pathways, playing a role in pathogenesis and mediating inflammatory processes through their proliferation (Smith, 2005). Thus, the analysed traits are expected to present a complex genomic architecture with several genes and pathways involved in their phenotypic expression. This is concordant with the possible overestimation of the SNP and regional effects due to the Beavis effect (Xu, 2003) as well as with the lack of consistency across QTLs detected in several studies (Buitenhuis et al., 2007; Scholey et al., 2012; Swalve et al., 2014; Malchiodi et al., 2018). Therefore, it is expected that increasing the sample size with additional accurate records will increase the accuracy of the estimates of these effects.

DCT is a novel trait analysed using genomic data for the first time in the present study. Previous studies have shown an association between lameness-related diseases such as sole ulcer and white line disease with a thinner digital cushion, indicating also a potential change in the tissue composition of the cushion (Bicalho et al., 2009). In our study, given the relatively small number of samples available, no significant or suggestive markers were identified. However, a significant genomic effect was detected for DCT at calving, providing a moderate genomic heritability of 0.23 ± 0.12. When compared with the heritability of 0.33 obtained in a previous pedigree-based study (Oikonomou et al., 2014), our estimate was smaller but within the standard error boundaries.

As with the lameness-associated lesions, the genomic architecture of DCT traits is expected to be polygenic, being particularly related with body fatty acid and lipid metabolism. Further studies with an increased sample size will refine the heritability estimates and provide some potential candidate genes associated with this structure.

## Conclusion

Four genomic regions were identified for digital dermatitis, interdigital hyperplasia, and sole haemorrhage, harbouring genes involved in inflammatory and fibroblastic processes. These traits are moderately heritable and potentially associated with a polygenic architecture. Therefore, the identification of associated regions may be useful to inform genomic selection programmes against lameness and to increase our knowledge of the underlying pathology.

In addition, this is the first study to address DCT from a genomic perspective, showing a moderate genomic heritability for this structure during the period of calving. The genomic architecture of this trait warrants further research attention.

## Data Availability Statement

The genotype data has been uploaded to a public repository hosted by the University of Edinburgh. Genotypes are therefore publicly available and can be obtained from: Edinburgh DataShare (University of Edinburgh), https://datashare.is.ed.ac.uk/handle/10283/3409.

## Ethics Statement

Ethical approval for the study was granted by the University of Liverpool Research Ethics Committee. ASPA regulated procedures were conducted under a Home Office License (Reference Number: PPL 70/8330).

## Author Contributions

ES-M analyzed the data and co-wrote the first draft of the manuscript. VB collected the data, performed laboratory work, analyzed the data, and co-wrote the first draft of the manuscript. RS provided funding and critically evaluated the manuscript. GO and GB secured funding, designed and supervised the study, and critically evaluated the manuscript.

## Funding

This study was funded by the Academy of Medical Sciences. VB acknowledges support from The Turkish Ministry of Education, and Ministry of Food, Agriculture, and Livestock. GO gratefully acknowledges support from the Wellcome Trust. GB and ES-M gratefully acknowledge funding from The Roslin Institute Strategic Programme (ISP) grants, and GB also the Rural and Environment Science and Analytical Services Division of the Scottish Government.

## Conflict of Interest

The authors declare that the research was conducted in the absence of any commercial or financial relationships that could be construed as a potential conflict of interest.

## Supplementary Material

Supplementary Figure 1 **Supplementary Figure 1 | (A)** digital dermatitis for farm records; **(B)** interdigital hyperplasia for farm records; **(C)** interdigital hyperplasia for combined records and; **(D)** sole haemorrhage for combined records]. Red lines correspond to the genome-wide significant threshold, and blue lines correspond to the genome-wide suggestive threshold.
